# Magnetic Composite Carbon from Microcrystalline Cellulose to Tackle Paracetamol Contamination: Kinetics, Mass Transfer, Equilibrium, and Thermodynamic Studies

**DOI:** 10.3390/polym16243538

**Published:** 2024-12-19

**Authors:** Pascal S. Thue, Alfred G. N. Wamba, Beatris L. Mello, Fernando M. Machado, Karoline F. Petroman, Willian Cézar Nadaleti, Robson Andreazza, Glaydson S. dos Reis, Mohamed Abatal, Eder C. Lima

**Affiliations:** 1 Environmental Science Graduate Program, Engineering Center, Federal University of Pelotas (UFPel), 989 Benjamin Constant St., Pelotas 96010-020, RS, Brazil; fernando.machado.machado80@gmail.com (F.M.M.); williancezarnadaletti@gmail.com (W.C.N.); robson.andreazza@ufpel.edu.br (R.A.); 2 Department of Process Engineering, Saint Jerome Catholic University Institute, Av. Akwa Koumassi, Douala BP 5949, Cameroon; ndialfred87@gmail.com; 3 Institute of Chemistry, Federal University of Rio Grande do Sul (UFRGS), Bento Gonçalves 9500, Porto Alegre 90010-150, RS, Brazil; beatrislisboa15@gmail.com (B.L.M.); karolinefariaspetroman@gmail.com (K.F.P.); 4 Graduate Program in Materials Science and Engineering (PPGCEM), Technological Development Center, Federal University of Pelotas (UFPel), Pelotas 96010-610, RS, Brazil; 5 Laboratory of Industrial Chemistry and Reaction Engineering, Faculty of Science and Engineering, Åbo Akademi University, 20500 Turku, Finland; glaydsonambiental@gmail.com; 6 Facultad de Ingeniería, Universidad Autónoma del Carmen, Ciudad del Carmen 24115, Mexico; mabatal@pampano.unacar.mx

**Keywords:** magnetic composite, adsorption, sustainable development goals, kinetics, mass transfer, equilibrium and thermodynamics

## Abstract

This study reported a one-spot preparation of magnetic composite carbon (MCC@Fe) from microcrystalline cellulose (MC). The pure cellulose was impregnated in iron (III) chloride solution and carbonized at 650 °C. The MCC@Fe composite adsorbent underwent various characterization techniques. XRD identified nanostructured Fe_3_O_4_ particles with an average crystallite size of 34.3 nm embedded in the core subunits of the material. FESEM images indicated a rough and irregular surface, with some cavities along its surface, incorporating Fe_3_O_4_ nanoparticles, while EDS analysis confirmed the presence of elements like Fe, C, and O. Notably, combining thermal and chemical treatments produces a composite with more pores and a high specific surface area (500.0 m^2^ g^−1^) compared to MC (1.5 m^2^/g). VSM analysis confirmed the magnetic properties (0.76 emu/g), while the Hydrophobic Index (*HI*) showed that MCC@Fe was hydrophobic (*HI* 1.395). The adsorption studies consisted of kinetic, mass transfer, equilibrium, and thermodynamics studies. Kinetic study of the adsorption of paracetamol on MCC@Fe composite proved to be rapid, and the time necessary for covering 95% of the surface (t_0.95_) was lower than 27 min following the fractal-like pseudo-first-order model (FPFO). Liu’s isotherm proved to be the most appropriate for understanding the adsorption equilibrium. Remarkably, the maximum sorption capacity (Q_max_) of paracetamol was 34.78 mg g^−1^ at 45 °C. The Δ*H*° value (+27.00 kJ/mol) and the negative Δ*G*° values were consistent with the physisorption mechanism and favorable process. Furthermore, the mass transfer mechanism showed that the transfer is governed by the intraparticle diffusion model, with surface diffusion being the rate-limiting step when considering the *Biot* number greater than 100. This research displayed a single-route production of inexpensive magnetic nano adsorbents capable of efficiently eliminating paracetamol from aqueous environments.

## 1. Introduction

The current understanding of water contamination levels with emerging substances highlights the urgent need for sustainable remediation methods that are both economically and environmentally viable. The increase in inappropriate effluent disposal has worsened surface and groundwater quality. Since water pollution seriously affects its availability, this phenomenon must be managed appropriately to reduce the impacts of increasing water scarcity. In 2022, the number of people around the world without access to drinking water reached 2.2 billion [1]. Since 2015, member countries of the United Nations (UN) have adopted the 2030 Agenda for Sustainable Development, presenting goals necessary to maintain life [2]. From this perspective, the UN established 17 Sustainable Development Goals (SDGs). Objectives 6, 11, and 14 are related to water quality and, consequently, the treatment of effluents to remove environmental contaminants [1].

Among contaminants of emerging concern (CECs), biologically active pharmaceutical compounds constitute a broad class of chemical substances with a significant environmental impact. They can be excreted by patients or disposed of inappropriately by users. Hospitals and pharmaceutical industries have also been identified as sources of these contaminants [3]. As they are resistant to inactivation and highly soluble in water, many CECs are not entirely removed by wastewater treatment plants. Consequently, these substances have been detected in surface water, groundwater, and drinking water around the world [4]. Although drugs are in low concentrations in the aquatic environment (ng L^−1^ to μg L^−1^), they still pose ecological and human health risks, as they are recalcitrant and biologically active [4]. The existence of traces of drugs in drinking water is a public health problem, as little is known about the potential long-term effects of ingesting drug mixtures. Therefore, its effective removal from water and aqueous effluents is a priority [5]. Paracetamol, also known as acetaminophen, is one of the most used analgesics worldwide [6]. Therefore, it is often found in drinking, surface, and water treatment plants [7].

Several approaches have been studied in search of the best treatment for removing drugs present in aqueous effluents, such as adsorption [8,9,10], electrochemical oxidation [11,12], biodegradation [13,14], Fenton processes [15,16], photocatalysis [17,18], photoelectrocatalysis [18,19], chemical oxidation [20,21], ozonization [22,23], and membrane filtration [24,25]. With the exception of adsorption, these advanced oxidative treatment methods practically suffer several drawbacks, such as the production of transformation by-products that could be even more toxic than the original pollutant [26,27]. In addition, the membrane filtration methods lead to the fouling of the membrane, decreasing the performance of the filtration method [28].

Conversely, adsorption is a promising procedure for water treatment because of its low investment, high performance, easy operation, and possibility of regeneration [29,30,31,32,33,34,35,36,37]. Furthermore, it does not generate toxic transformation products [26,27]. The adsorbate (micropollutant) is transferred from the aqueous solution to the adsorbent surface, diminishing the living beings’ exposure to these substances. Afterward, the treated effluent can be liberated safely into water bodies or reused for some specific industrial application [38,39,40,41,42].

Carbon-based materials are the main employed adsorbents for wastewater treatment because of their high surface area, higher total pore volume, suitable structures of pores, and high affinity for the uptake of organics [40,41,42]. Among the carbonaceous adsorbent materials are activated carbon [40,41,42,43,44], carbon nanotubes [45], graphene materials [46,47,48,49], biochars [50,51,52,53,54,55], and composite materials with carbon-based materials in their constitution [56,57,58,59,60,61]. Many carbon-based materials are prepared using waste biomass as a carbon source [40,41,50,51,52,53,54,55,56,57,58,59]; conversely, the use of pure microcellulose could lead to a more homogeneous carbon material than waste biomass [62]. Pyrolysis of microcrystalline cellulose can produce biochar rich in micropores and mesopores structures, thus increasing its performance in removing organic compounds [62,63,64].

Despite the potential of using microcellulose for preparing carbon-based materials [62,63,64], there is a significant gap in the literature regarding its application for pharmaceutical removal from aqueous effluents.

One primary limitation of powdered adsorbents is their separation from the aqueous medium after the adsorption operation [33,37,39]. Typically, the loaded adsorbent needs to be centrifuged [33,37,39] before measuring the remaining organic compound in the effluent solution. Conversely, magnetic adsorbents offer a valuable method for separating the loaded adsorbent from the wastewater [65,66].

Magnetic biochar is a composite material that has attracted increasing attention as it allows separation to be carried out by applying an external magnetic field [67,68]. The incorporation of metals with magnetic properties also promotes their reuse and prevents losses of the adsorbent [65,66,69]. Thus, studies have been carried out to investigate the addition of magnetic properties to these adsorbents to facilitate their separation after adsorption [65,66,67,68,69]. Conversely, a majority of magnetic carbon composite materials are prepared in multi-steps, making it more challenging to obtain this magnetic adsorbent [70,71].

The objectives of this research are (1) the production of a magnetic composite carbon (MCC@Fe) in a single-pyrolysis route process between microcrystalline cellulose (MC) and FeCl(III) solution; (2) the application of the MCC@Fe composite in the paracetamol uptake from aqueous wastewater; and (3) the explanation of adsorption mechanism through kinetic, isotherm, mass transfer, and thermodynamic studies. The pure MCC was mixed with FeCl_3_ solution, forming one paste that was furtherly carbonized at 650 °C to produce MCC@Fe magnetic composite carbon. The composite was characterized by FTIR, scanning electron microscopy (SEM/EDX), elementary chemical analysis CHN/O, XRD, pH_pzc_, TGA/DTA, and magnetometry analysis (VSM). Then, the material was used to conduct the batch adsorption of paracetamol. The mechanisms involved in the adsorption process were elucidated using kinetic and equilibrium models and mass transfer, and the thermodynamic parameters were determined. The best experimental conditions were applied, thus obtaining the maximum adsorption capacity of the material.

## 2. Experimental

### 2.1. Materials

Paracetamol synthetic solutions were prepared with deionized water. The molecule was used as a source of pharmaceutical contaminant provided by Merck and used without further purification. The physicochemical property can be found in Supplementary Figure S1. The iron (III) chloride salt, FeCl_3_, was purchased from Neon (São Paulo, Brazil). This reagent was used as an activating agent and to provide magnetic properties to the material. Sodium hydroxide and hydrochloric acid solutions were used to adjust the pH. Mingtai Industry, Taiwan, provided microcrystalline cellulose. It was used without further treatment to prepare the magnetic composite carbon (MCC@Fe). The other reactants used were analytical grade and were not purified beforehand.

### 2.2. One-Spot Preparation Process of Magnetic Composite from Microcrystalline Cellulose-MCC@Fe

The magnetic carbon composite was produced by a single-route process, as described below. FeCl_3_ was used as an activating agent to improve textural properties, precisely pore size and specific surface area, and to provide magnetic properties to the material. First, FeCl_3_ salt was dissolved in deionized water in order to have a 0.62 mol/L solution. Afterward, 10 g of the pure microcrystalline cellulose powder (φ < 100 µm) was added to the solution and mixed thoroughly to form a paste. The mixtures were stirred up with a glass stick at 90 °C for two hours [50,51,52]. Then, the homogeneous pastes formed were oven-dried at 105 °C overnight and carbonized in a conventional furnace (Sanchis, Brazil) [50,51,52]. The heating was carried out from 25° to 650 °C, at a heating rate of 10 °C min^−1^, under N_2_ atmosphere (flow rate of 12 L h^−1^). After reaching 650 °C, the temperature was maintained for 60 min. The furnace was then shut down. However, the nitrogen stream was kept until the temperature reached <200 °C [33,65,66]. It is worth reminding the reader that the magnetic property of the material was developed during the carbonization, where FeCl_3_ is converted to Fe_3_O_4_. After cooling, the obtained materials were refluxed with 0.1 mol/L HCl solution at 80 °C for 120 min. Then, the carbon material was thoroughly washed with deionized water until the pH values of the washing waters attained pH 6.0 [33,41]. Finally, the materials were oven-dried at 105 °C overnight [65,66]. The magnetic biochar cellulose was labeled MCC@Fe.

### 2.3. Characterization of *Magnetic Composite Carbon (MCC@Fe) Adsorbent*

The characterization of MCC@Fe adsorbent was achieved using various analytical techniques. The characterization of pure microcellulose was also made for comparison. Elemental analysis was conducted to determine carbon, nitrogen, and hydrogen content, with oxygen mass fraction calculated from thermogravimetric analysis (TGA). X-ray diffraction (XRD) was used to evaluate the crystallinity of both raw cellulose and MCC@Fe, while the average crystallite size of Fe_3_O_4_ particles was determined using the Scherrer model. Surface morphology was examined via scanning electron microscopy (SEM), and functional groups were analyzed with Fourier-Transform Infrared Spectroscopy (FTIR). The chemical composition was further analyzed using energy-dispersive X-ray spectroscopy (EDS). Nitrogen adsorption/desorption measurements were used to assess the textural properties, and thermal stability was studied using TGA/DTG. Hydrophilic/hydrophobic behavior was measured over 24 h at 25 °C, and magnetic properties were assessed using a Vibrating Sample Magnetometer (VSM). More details about the condition are given in the Supplementary Materials [37,39,40,45,50,51,52,65,66,72,73].

### 2.4. Adsorption Studies

In order to test the effectiveness of the magnetic composite carbon (MCC@Fe), adsorption experiments were carried out in a batch reactor, using paracetamol (PCT) as a pollutant [39,40,41,42,45]. Preliminary adsorption experiments were conducted to ensure reproducibility, reliability, and accuracy. A 20 mL solution of paracetamol (PCT), with concentrations ranging from 10 to 500 mg/L, was mixed with 30 mg of MCC@Fe carbon material in 50 mL Falcon tubes. The pH ranged from 2.0 and 10.0. The tubes were placed in a thermostatic reciprocating agitator and shaken at varying times (1–300 min) and temperatures (10–45 °C), with a shaking speed of 150 rpm. After the process, the solid phase was separated from the liquid phase. The remaining unadsorbed PCT in the liquid phase was measured using a spectrophotometer at a wavelength of 257 nm. More details are found in the Supplementary Materials [39,40,41,42,45,74,75,76,77,78,79,80].

## 3. Result and Discussion

### 3.1. X-Ray Diffraction Analysis of Cellulose Microcrystalline (MC) and MCC@Fe

**Figure 1:**
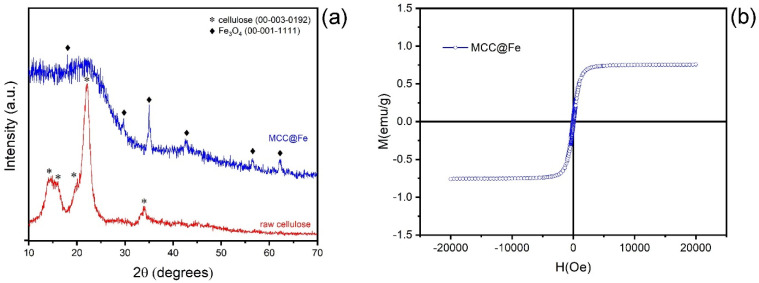
(**a**) Diffractograms of raw CP (red line) and MCC@Fe adsorbent (blue line). (**b**) VSM hysteresis loops of MCC@Fe at room temperature.

Figure 1a presents the diffractograms of both raw cellulose and MCC@Fe. In the MC pattern, characteristic peaks of the cellulose structure (ICCD 00-003-0192) are observed at approximately 14.1°, 16.3°, 19.7°, 22°, and 34°, associated, respectively, with diffraction planes of (101), (10$\bar{1}$), (021), (200), and (040) [81]. The MCC@Fe reveals a distinct “slender hump” until approximately 32°, which is typical of carbon amorphous material. Furthermore, the MCC@Fe diffractogram indicates the presence of Fe_3_O_4_ (magnetite, cubic crystal system, ICCD 00-001-1111), as evidenced by diffraction peaks at 18.1°, 30°, 35.3°, 43°, 57°, and 62.6°, corresponding to (111), (220), (311), (400), (511), and (440) reflections, respectively. The Scherrer equation further confirms that a single-step production route allows for the synthesis of nanostructured Fe_3_O_4_ particles with an average crystallite size of 34.3 nm [72]. Therefore, the XRD of the MCC@Fe particles shows that the iron metal embedded in its structure has crystalline nanometric size.

### 3.2. Magnetic Features

The magnetization curve and the values of the hysteresis parameters of the MCC@Fe adsorbent are shown in Figure 1b. The magnetization curve was obtained at room temperature. The result indicates that MCC@Fe presents values of coercivity (H_C_ = 86.79 Oe) and remaining magnetization (M_r_ = 0.0614 emu g^−1^) close to zero, suggesting a superparamagnetic material. In addition, the saturation magnetization (M_S_) value was 0.76 emu g^−1^, which is comparable with some magnetic biochar materials [82,83]. For instance, Zhu et al. [82] obtained a magnetic biochar prepared via simultaneous magnetization and activation, presenting a saturation magnetization of 0.76 emu g^−1^. Similarly, Dong et al. [83] reported a magnetic biochar from three types of biomass feedstocks prepared via precipitation method and carbonization at 700 °C, with an M_S_ value of 1.45 emu g^−1^. The lower value of M_S_ of the MCC@Fe adsorbent can be attributed to the presence of graphitic layers or amorphous carbon that might lead to the encapsulation of Fe_3_O_4_ nanoparticles in carbon core–shell subunits [84].

### 3.3. Textural and Elemental Analysis

The textural and CHN/O elemental analysis of the cellulose [39] and MCC@Fe composite material are presented in Table 1. The BET surface areas of the cellulose (1.5 m^2^ g^−1^) and MCC@Fe composite (500.0 m^2^ g^−1^) were obtained. This result shows that, after combining chemical treatment with iron (III) chloride and subsequent pyrolysis, the carbon composite has a remarkable increase in the specific surface area and also presents magnetization. Therefore, in addition to providing magnetic properties, FeCl_3_ can affect the textural characteristics of the material. We must also take into account that MCC@Fe showed nanostructured Fe_3_O_4_ particles with an average crystallite size of 34.3 nm in its structure (see discussion in Section 3.1). Based on the observations, the obtained value of BET surface area is high.

Additionally, Table 1 shows a difference in the chemical contents of C, H, and O of the samples. When the MCC@Fe is fabricated, it is possible to observe a massive increase in carbon content while oxygen and hydrogen contents decrease considerably. This behavior was expected. In fact, according to the literature [50,51,52,65,66], a higher carbon content after pyrolysis indicates a progressive formation of aromatic compounds (polycondensation and aromatization) in the composite structure after carbonization in the presence of metallic components [50,51,52,65,66]. Indeed, during pyrolysis, metals such as iron act as dehydrating agents and form iron oxides and, where appropriate, iron oxide nanocrystals, as presented in Section 3.1. Losses of oxygen and hydrogen contents are attributed to the cleavage and cracking of poorly oxygenated particles of microcrystalline cellulose [85]. Oxygen and hydrogen are mainly eliminated in the form of CO_2_ and H_2_O during the carbonization process. In fact, as the temperature in the system continues rising, the thermal decomposition of the cellulose is rapidly carried out, and a large number of decomposition products, such as CO_2_, CO, H_2_, and C_1_-C_4_ gases, are produced.

The Hydrophobicity Index is defined by the following equation [73]:

(1) HI=quantity of n−heptane vapor (mg)mass of adsorbent (g)quantity of water vapor (mg)mass of adsorbent (g)

The obtained value of the *HI* is 1.395, which can be considered hydrophobic when compared to the pristine cellulose [39,73]. The hydrophobic character of the material means that the adsorbent surface presents a great trend for adsorbing molecules that are hydrophobic. Therefore, the thermal and chemical treatment allows for the production of a magnetic hydrophobic material, while pure cellulose is highly hydrophilic (*HI* of 0.318) [39]. These results are consistent with the C, H, and O contents of both materials. In fact, higher carbon content in activated carbon is commonly linked with the hydrophobic property, while higher oxygen content is related to the hydrophilic property [39,73]. The carbonization led to a more aromatic structure of the material during the polycondensation and aromatization stages. This might elevate the possible interactions of the paracetamol and the adsorbent through hydrogen bonding, electrostatic attractions, and π-π stacking of the ring of the paracetamol molecule and the ring of MCC@Fe magnetic material.

### 3.4. SEM and EDS MCC@Fe

**Figure 2:**
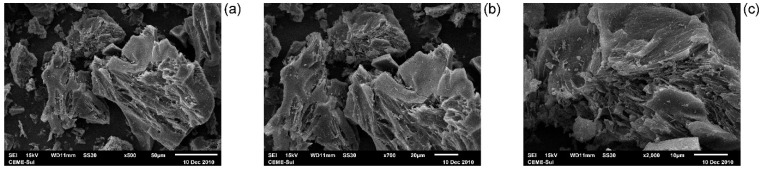
SEM images of MCC@Fe adsorbent at different magnifications: (**a**) ×500 (**b**) ×700 and (**c**) ×2000.

SEM achieves the morphological analysis of MCC@Fe magnetic material. The images were taken at augmentations of ×500, ×700, and ×2000 to appreciate very well the quality of the information and are presented in Figure 2. It is possible to notice on the SEM images that the MCC@Fe magnetic composite had a rough and irregular surface and some cavities along its surface. The cavities and channels are of different sizes. Overall, the SEM photographs show that FeCl_3_ and thermal treatment can be used to improve the physicochemical properties of the carbon material. As previously mentioned, such cavities are formed during the activating process, which involves chemical and thermal treatment. Furthermore, these cavities and channels enable the application of the carbon material in adsorption processes because they facilitate pollutants such as paracetamol species to enter the channels of the adsorbent until reaching the pores of minor dimensions [50,51,52]. This statement is discussed further in Section 3.8, which discusses the study of mass transfer.

The chemical composition of the surface of MCC@Fe magnetic material was obtained by energy-dispersive X-ray spectroscopy (Supplementary Figure S2). The results show values of carbon (62.31%), oxygen (14.66%), and iron (2.81%). The carbon and oxygen contents shown by EDX are slightly lower compared to the CHN/S elemental analysis because EDX analysis is semiquantitative. Notwithstanding, they do lead to the same conclusion when compared to the pure cellulose before the thermochemical treatment. In addition, the peak related to iron matches the XRD and magnetism results discussed in the previous sections, which show the presence of Fe_3_O_4_ nanoparticles in carbon core–shell subunits. The iron content (2.81%) matches with the ash content (2.73%) obtained by TGA analysis. This is further discussed in Section 3.7.

### 3.5. Acidity Property of the Magnetic MCC@Fe Adsorbent

The pH_pzc_ is an important surface parameter for evaluating the charge developed in the solid when it is present in an aqueous solution with a determined pH. The pH_pzc_ is very important when the primary mechanism is governed by electrostatic interaction. Supplementary Figure S3 shows that the pH_pzc_ of the magnetic MCC@Fe was 4.803, meaning that the global charge of MCC@Fe is positive when the pH < 4.803, and the global charge is negative when it is higher. The adsorption experiment in this study was conducted at pH 7, chosen as the optimal condition, implying a negatively charged surface characteristic of the material. This observation can be attributed to the presence of oxygenated groups susceptible to protonation in aqueous media, resulting in an acidic surface material [50,51,52]. Additionally, paracetamol’s molecular structure has a phenol group (-OH), exhibiting weak acidic properties, with a pKa of 9.46 (Supplementary Figure S1).

Consequently, paracetamol predominantly exists in its neutral, protonated form at pH 7, indicating that magnetic MCC@Fe demonstrates enhanced adsorption capability toward unionized paracetamol molecules compared to their ionized counterparts. This underscores the idea that diminished electrostatic attraction as a favorable or primary mechanism in the adsorption process. The adsorption mechanism is further discussed in Section 3.10.

### 3.6. FTIR Magnetic MCC@Fe

**Figure 3:**
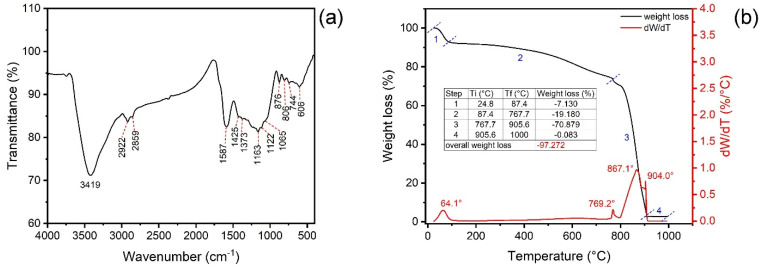
(**a**) FTIR spectra and (**b**) TGA/DTG curves of MCC@Fe magnetic composite.

Figure 3a highlights the FTIR spectra of magnetic MCC@Fe adsorbent material. The bands at 3419 cm^−1^ can be assigned to the stretching vibrations of the O-H group from phenols, alcohols, or carboxylic acids present on the surface of the MCC@Fe magnetic composite. It can also be attributed to the presence of adsorbed water in the material. The CH_2_ stretching bands at 2922 and 2858 cm^−1^ are attributed to the asymmetric and symmetric stretching of CH_2_ groups [45,86] or methoxy group attached to an aromatic ring [45], respectively. The aromatic ring modes appear at 1587 and 1425 cm^−1^ [65,66,86]. The band at 1373 cm^−1^ could be ascribed to the O-H bending [41,86].

Moreover, a band at 1163 and 1122 cm^−1^ can be found on the spectra and can be assigned to the C–O stretching vibrations of esters [40,86]. The band at 1065 cm^−1^ is assigned to the C–O stretching of phenol, ester, or carboxylic acid [37,86]. The bands at 876 and 806 cm^−1^ are ascribed to the out-of-plane C–H bending of aromatics [50,86]. In addition, bands of Fe-O stretching vibration could be found at 606 and 744 cm^−1^, confirming the presence of Fe_3_O_4_ nanoparticles embedded in the bulk of the MCC@Fe magnetic composite, as already mentioned in the previous section [65,66].

Therefore, the magnetic composite adsorbent shows a wide variety of functional groups, such as –OH, –COO-, and arene rings, that might interact with paracetamol species in the adsorption process through hydrogen bonds, π-π interaction*,* n-π interaction, and d-π interaction.

### 3.7. TGA Analysis and Thermal Stability of MCC@Fe Adsorbent

The thermal stability behavior and mass loss of MCC@Fe magnetic adsorbent were explored using thermogravimetric analysis. The thermogram is presented in Figure 3b. The analysis was performed in a single run, with two different atmospheres, from room temperature to 800 °C (N_2_ flow) and from 800° to 1000 °C (synthetic air atmosphere) [39,50,51,52]. The air atmosphere allows one to obtain the ash content, representing the inorganic part of the material [39,50,51,52]. The thermogravimetric curves can be divided into four regions of weight loss. The weight loss in the first area is 7.13% (24.8–87.4 °C) and is associated with the loss of adsorbed water [39,65,66]. The weight loss in the second region is 19.18% (87.4.–767.7 °C) and is due to bounded water and hydroxyl groups in the magnetic composite [39,65,66]. The main weight loss occurs in the third region, attaining a loss of 70.18% (767.7–905.6 °C) when the inert atmosphere is switched to oxidizing atmosphere [39,50,51,52]. It is remarkable to observe that the material is very stable until 800 °C, which can be very interesting for comprehensive applications. The weight loss in this third region is assigned to the carbonaceous matrix degradation [39,65,66]. In the fourth region, the weight loss was 0.083% (905.6–1000 °C), which is the final destruction of the carbon matrix, achieving the inorganic ashes [39,65,66]. In total, 2.73% of the ash content was left after thermal decomposition in the presence of air [39,65,66]. As the unique inorganic used in the preparation of the MCC is Fe_3_O_4_, it is possible to state that the obtained value of the ash content refers to the Fe_3_O_4_ fraction in the structure of the MCC@Fe adsorbent.

### 3.8. Adsorption Kinetics

**Figure 4:**
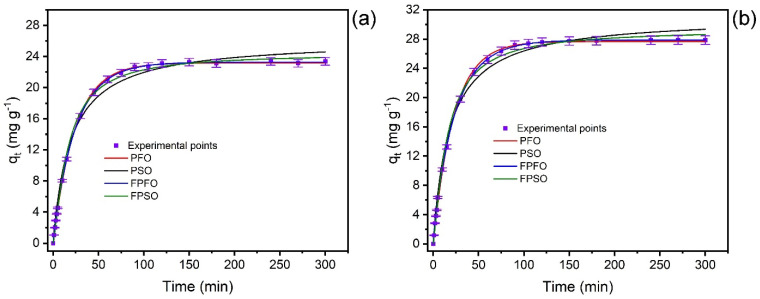
Kinetics curves at (**a**) 125.0 mg/L and (**b**) 250.0 mg/L, using MCC@Fe composite adsorbent (pH 7.0, 1.5 g L^−1^ of adsorbent dosage, and 25 °C).

The paracetamol kinetics uptake on the MCC@Fe composite was carried out, and the kinetic model parameters (Table 2) and curves (Figure 4a,b) were obtained. The values of the statistical parameters adjusted determination coefficient (*R*^2^_adj_), standard deviation of the residues (SD), and Bayesian Information Criterion (BIC) were utilized to obtain the best-fitted kinetic model. The best-fitted physical model will present the following characteristics: values of *R*^2^_adj_ closer to 1.00, the lowest SD, and BIC values [74]. Table 2 demonstrates that the FPFO kinetic model has these statistical parameters described above for both concentrations, suggesting that FPFO well described the kinetic adsorption of PCT molecules on the MCC@Fe adsorbent. Another statistical tool that could undoubtedly affirm what is the best-fitted model is the BIC [39,74]. When the ΔBIC is >10, certainly, the model with the lowest BIC value is the best-fitted model [39,74]. The ΔBIC between PFO and PSO and for FPSO vs. FPFO ranged from 28.18 to 92.84 (C_o_ 125.0 mg L^−1^) and from 27.53 to 67.91 (C_o_ 250 mg L^−1^) (see Table 2). These ΔBIC values confirm that the FPFO is the best-fitted kinetic model [39,74].

The half-lives (t_1/2_), which correspond to the time to achieve 50% saturation of the adsorbent, were 11.63 min (125.0 mg L^−1^) and 13.94 min (250 mg L^−1^). This result suggests fast adsorption of PCT molecules in the very first moments of the experiment (Figure 4a,b). At t_0.95_, which corresponds to the time by which 95% of saturation of MCC@Fe is achieved, the values are 22.10 min (125 mg L^−1^ PCT) and 26.49 min (250 mg L^−1^ PCT). Additionally, it is possible to see both in Figure 4a,b, and Table 2 that as the concentration of the PCT molecules is increased in the solution, the equilibrium time also increases, suggesting that a high concentration might lengthen the equilibrium time. For subsequent experiments, the contact time was fixed at 60 min, as the PCT concentration ranged from 20 to 500 mg L^−1^.

**Figure 5:**
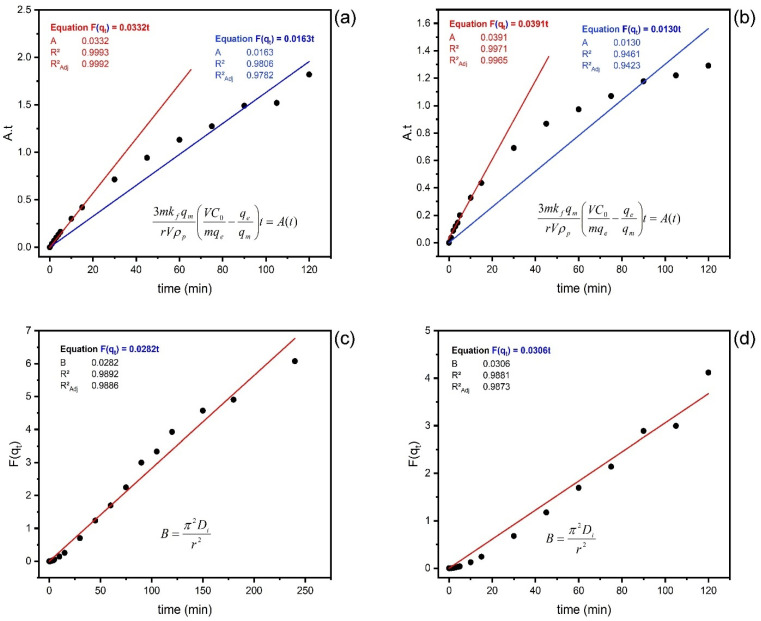
(**a**,**b**) Film diffusion model. (**c**,**d**) Intraparticle diffusion model of adsorption of paracetamol on MCC@Fe composite. Initial pH at 7, temperature at 25 °C, initial concentrations at 125 mg/L (**a**,**c**) and 250 mg/L (**b**,**d**), and dose (**d**) adsorbent at 1.5 g/L.

To better understand the adsorption process of paracetamol onto MCC@Fe, linear models of film diffusion and intraparticle diffusion were applied to experimental data of paracetamol adsorption kinetics. Figure 5 presents the fitting results of the two diffusion models of the experimental data, describing the relationship between the dimensionless numbers, ***At*** (constant proportional to the transfer coefficient in the film) and ***Bt*** (constant proportional to the transfer coefficient by intraparticle diffusion), with the contact time (adsorbent–adsorbate). It is possible to notice that the theoretical line (***At***) describing the film diffusion does not fit with the experimental points as the adsorption time increases for both concentrations (blue line). The plots of ***At*** versus *t* (red line) show linear relationships passing through the origin (0,0) observed for the early (5–10 min) adsorption stage (corresponding to q_t_ from 0 to about 0.3 q_e_). This means that film diffusion is controlling the first stage of adsorption. Afterward, the curves deviate from linearity, suggesting that another mechanism occurred. The values of parameter *A* are, respectively, 0.0332 min^−1^ and 0.0391 min^−1^ for both initial concentrations of paracetamol, 125 and 250 mg/L.

Furthermore, the theoretical line (*Bt*) describing the intraparticle diffusion agrees with the experimental data (R^2^ = 0.9913 and 0.9939) near the equilibrium adsorption stage (corresponding to q_t_ from 0 to about 0.85 q_e_). The values of ***B*** are 0.0414 min^−1^ and 0.0427 for 125 mg/L and 250 mg/L, respectively. This result is achieved for a surface of the adsorbent coverage rate of less than 85%, leading to the approximation of the Weber and Morris model [78]. However, no significant difference is observed for a recovery rate greater than 85% of the adsorbent using the modified diffusion model [87] (see Equation (S24) in the Supplementary Materials). The values of ***B*** are 0.0412 min^−1^ and 0.0422 for 125 mg/L and 250 mg/L, respectively. Thus, the adsorption process of paracetamol is governed by intraparticle diffusion. Additionally, the *Biot* number (*Bi*) greater than 100 confirms this statement (Table 3). In fact, *Bi* > 100 means that the adsorption process is controlled by intraparticle transfer through diffusion on the surface of the adsorbent [87,88]. Knowing the molecular radius of paracetamol obtained by MarvinSketch software (Supplementary Figure S1), it is possible to state that the molecules will hardly diffuse inside the micropores; instead, they will diffuse either in the macropores or in the mesopores. This result also allows one to conclude that surface adsorption is the rate-limiting step in the adsorption process of paracetamol onto the MCC@Fe composite.

### 3.9. Equilibrium Studies

**Figure 6:**
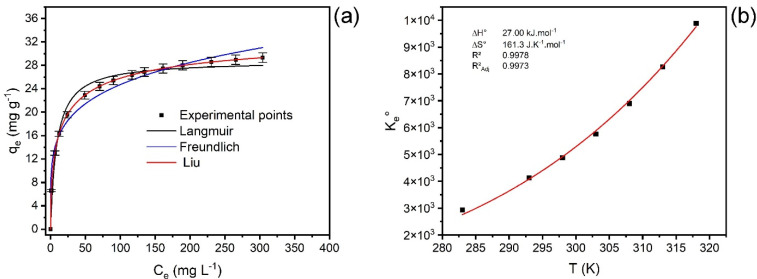
(**a**) Adsorption isotherm of uptake PCT onto MCC@Fe adsorbent at 45 °C. (**b**) Van’t Hoff graph for uptake of PCT onto MCC@Fe adsorbent. Conditions: 1.5 g L^−1^ of adsorbent dosage; contact time, 60 min; and pH 7.

The isothermal data were fitted using Langmuir, Freundlich, and Liu equilibrium models. The equilibrium experiments were realized from 10 °C to 45 °C. Figure 6a and Table 4 show the PCT uptake isotherms onto the MCC@Fe adsorbent at 45 °C and 10–45 °C, respectively. As expected, the adsorbed quantity at the equilibrium (q_e_) increased as the concentration of the PCT molecules also increased till attaining the equilibrium where no significant changes are observed when increasing the concentration of the PCT in solution. The first observation can be explained by the fact that when the initial concentration increases, more PCT molecules are available to be adsorbed onto the active sites of the adsorbent.

Moreover, the diffusion of the PCT in solution at high concentrations is much higher than at low concentrations [33,37]. As the adsorbate concentration augments, the resistance to the PCT mass transfer between the adsorbent and the liquid phase is diminished; also, the distance between PCT molecules and the adsorbent is shortened.

Based on *R*^2^_adj_ and SD values, the Liu isotherm model is the one that best describes the equilibrium data of adsorption of PCT molecules onto MCC@Fe composite at all experimental temperatures (Table 3). Liu models suggest that the saturation of the MCC@Fe composite is predicted, the multilayers of the PCT molecules over the adsorbent could happen, and the activated adsorption sites of the adsorbent can have different energies [74]. The values of the standard deviation of the residues (SD) of the Langmuir, Freundlich, and Liu models range from 0.04809 to 1.326, from 0.7760 to 3.177, and from 0.004057 to 0.1978, respectively. The lowest values of the Liu isotherm model mean that the predicted theoretical equilibrium adsorption capacity (q_i,model_) is closer to the experimental equilibrium adsorption capacity (q_i,experimental_). Additionally, the *R*^2^_adj_ values close to 1 suggest that the Liu isotherm model is the best-fitted isotherm model.

On the other hand, to confirm the SD values, after applying the variation in ΔBIC between Langmuir and Liu and ΔBIC between Freundlich and Liu, all values are much higher than ten at all temperatures [39,74]. Therefore, it is possible to conclude that the Liu model is undoubtedly the best-fitted model describing the uptake of PCT molecules onto MCC@Fe material. Table 4 shows that the temperature slightly affects the adsorption capacity of the material. In fact, as the temperature enhanced from 10 to 45 °C, the Q_max_ also augmented from 22.74 to 34.78 mg/g, the latter being the highest adsorbed amount of PCT molecules onto MCC@Fe composite.

### 3.10. Thermodynamics Studies: Mechanism of Adsorption and Regeneration

For thermodynamic studies, the Liu equilibrium constant (K_g_), which was the best-fitted model to explain the equilibrium data from 10 to 45 °C, was utilized to calculate the thermodynamic equilibrium constant ($K_{e}^{0}$) according to the previous study [80]. The latter parameter is essential to find the enthalpy and entropy of the adsorption and conclude if the adsorption process is favorable or not. The values of ∆*H*°, ∆*S*°, and ∆*G*° of the adsorption of PCT on MCC@Fe are reported in Table 5.

The Gibb’s free energy change (∆*G°*) values obtained are all negative (see Table 5), while the enthalpy change (∆*H°*) is positive (see Figure 6b). These thermodynamic results imply an endothermic and favorable adsorption process.

It is possible to observe in Table 5 that the value of Δ*H*° was <30 kJ mol^−1^, whose value is compatible with physisorption [89]. Moreover, the positive values of ∆*S°* suggest that increasing temperature also increases randomness during the adsorption process of PCT onto MCC@Fe at the adsorbent/liquid interface [74,80]. The *R*^2^*_adj_* values of the nonlinear plots of the van’t Hoff equation (*K_e_*^0^ versus *T*) (See Figure 6b) is 0.9973 for the adsorption of paracetamol. Based on the obtained results, the physical adsorption of paracetamol (PCT) onto the MCC@Fe composite occurred through several mechanisms: pore-filling, hydrogen bonding, π-π interaction, n-π interaction, and d-π interaction, each contributing to the overall efficacy of the adsorption process.

Pore-filling occurs as PCT molecules migrate into the interconnected pores of the adsorbent material via mass transfer. Activated biochar, characterized by its porous structure, provides a network of interconnected voids where PCT molecules can reside, thereby facilitating their adsorption.

π-π interaction is evident due to the planar structure and conjugated π-electron systems of polycyclic aromatic hydrocarbons like PCT. This interaction, also known as π-π stacking or π-complexation, occurs when the delocalized π-electrons of PCT align with the π-electron clouds of the adsorbent material, leading to attractive forces that promote adsorption.

Hydrogen bonding also contributes to the adsorption process, whereby PCT molecules form hydrogen bonds with electronegative atoms (e.g., oxygen) present in the adsorbent material. These interactions enhance the overall adsorption capacity by creating additional attractive forces between PCT and the adsorbent surface.

Additionally, n-π interaction, or nucleophile–π interaction, involves non-covalent attractive interactions between lone pair electrons of nucleophilic atoms (e.g., nitrogen and oxygen) and the π-electrons of aromatic ring systems in composite carbon. This interaction further stabilizes the adsorption of PCT molecules onto the adsorbent surface.

**Figure 7:**
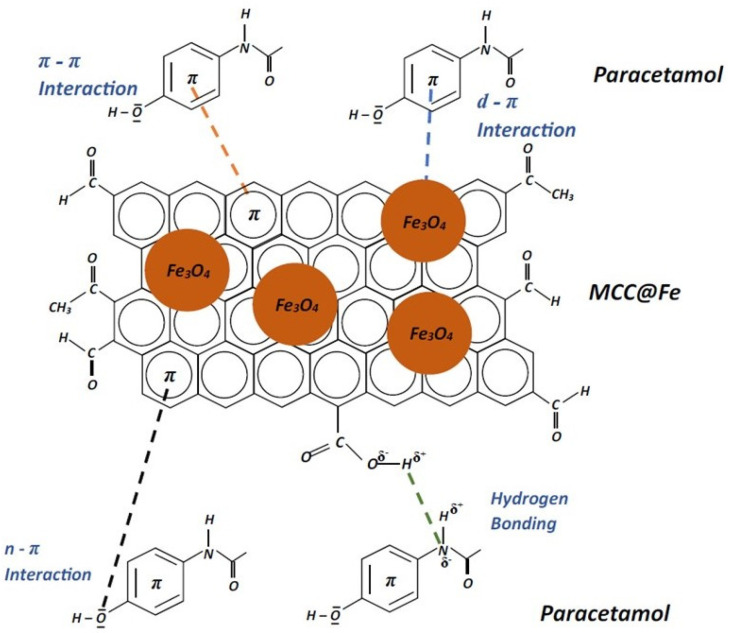
Possible mechanism of interaction of PCT onto MCC@Fe.

A potential d-π interaction can be elucidated by examining the mechanism of the corrosion-inhibition effect. Specifically, PCT molecules can form a protective layer that prevents the metal sites from undergoing oxidative reactions with the surrounding medium. In this context, the interaction occurs between the LUMO orbitals of iron and the non-bonding or π electrons of the inhibitor molecules, thereby confirming that PCT molecules can be strongly adsorbed onto the Fe_3_O_4_ nanoparticles within the MCC@Fe composite [90]. Figure 7 illustrates possible interactions between PCT molecules and MCC@Fe surfaces.

Overall, the adsorption kinetics included three processes: (1) liquid film diffusion, (2) pore diffusion, and (3) physical adsorption.

### 3.11. Regeneration of the MCC@Fe Composite Material

**Figure 8:**
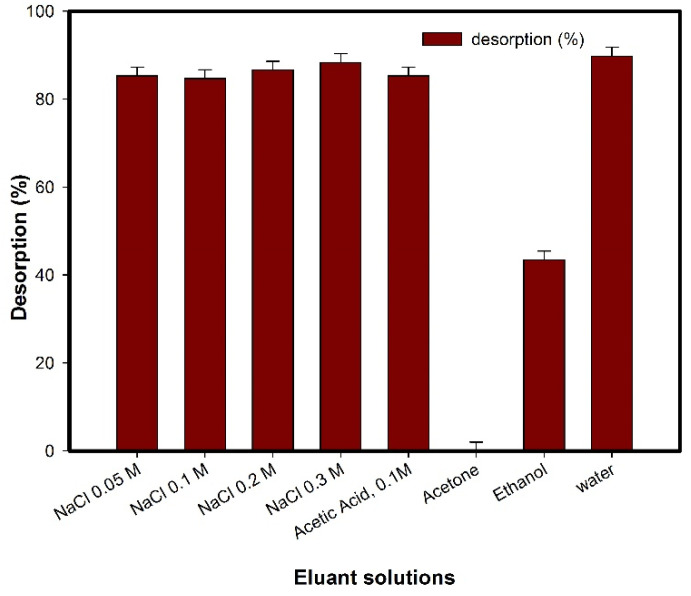
Desorption experiments of MCC@Fe composite for PCT.

In addition, the regeneration experiment was carried out after the adsorption of PCT onto the MCC@Fe composite. Several sodium chloride solutions at concentrations ranging from 0.05 to 0.3 M, organic solvents (ethanol and acetone), acetic acid 0.1 M, and water were used for the desorption experiment of PCT molecules from the MCC@Fe surface. Figure 8 shows the desorption percentage after the adsorption process. It is possible to see that water showed almost 90% of desorption. However, sodium chloride solution also shows a significant percentage of desorption. This result further confirms that the interaction between adsorbent and adsorbate was mainly physical, as discussed previously, meaning a weak interaction. Therefore, the desorption process guarantees the potential application of the MCC@Fe composite material in industrial wastewater treatment.

## 4. Conclusions and Final Remarks

In this study, we developed a magnetic composite carbon (MCC@Fe) from microcrystalline cellulose (MCC), using iron (III) chloride and carbonizing at 650 °C. The material was characterized and tested for paracetamol (PCT) adsorption. XRD showed magnetite (Fe_3_O_4_) in the composite, with an average crystallite size of 34.3 nm, giving the material magnetic properties. The surface area of MCC@Fe (500 m^2^/g) was significantly higher than pure cellulose (1.5 m^2^/g), indicating improved porosity and surface structure after FeCl_3_ and thermal treatment.

The MCC@Fe adsorbent shows a wide variety of functional groups, such as –OH, –COO-, arene rings, and bands of Fe-O stretching that might interact with paracetamol species during the adsorption process by hydrogen bonds, π-π interaction*,* n-π interaction, d-π interaction, and van der Waals forces.

The kinetic data better fitted the FPFO, and 95% of the adsorbent sites were saturated in less than 27 min, showing quick adsorption of PCT molecules. The equilibrium study reveals that increasing the temperature and the concentration of PCT in the solution will also increase the adsorption capacity. The Liu isotherm model best fits the data, indicating multilayer adsorption. The maximum adsorption capacity (Q_max_) was 34.78 mg/g at 45 °C.

Mass transfer analysis revealed that film diffusion controlled the initial stage of adsorption, while intraparticle diffusion governed the later stages. The process involved liquid film diffusion, pore diffusion, and physical adsorption, with surface diffusion being rate-limiting. This work demonstrates the possibility of producing magnetic composite carbons with a relatively low concentration of iron (III) chloride from microcrystalline cellulose for removing paracetamol, an emerging contaminant concern frequently detected in water.

## Figures and Tables

**Table 1:** Cellulose and MCC@Fe textural and CHN/O elemental analysis.

Sample	Particle Size (µm)	S_BET_ (m^2^ g^−1^)	%C	%H	%O	Ash	*HI*	pH_pzc_
Cellulose	≤100	1.50	41.78	6.05	51.17	0.00	0.318	6.52
MCC@Fe	22	500.0	75.33	2.97	18.97	2.73	1.395	4.803

**Table 2:** Kinetic parameters for paracetamol uptake onto MCC@Fe material. Conditions: 30.0 mg MCC@Fe, 20.00 mL of PCT solution, 25 °C, and pH 7.

	Paracetamol (PCT)	
Pseudo-first order	**125 mg L^−1^**	**250 mg L^−1^**
q_e_ (mg g^−1^)	23.16	27.68
k_1_ (min^−1^)	0.0415	0.04379
t_1/2_ (min)	11.58	13.84
t_0.95_ (min)	22.00468	26.29
*R* ^2^_adj_	0.9997	0.9990
SD (mg g^−1^)	0.1696	0.3480
BIC	−64.097	−35.340
Pseudo-second order		
q_e_ (mg g^−1^)	26.14	31.09
k_2_ (g mg^−1^ min^−1^)	0.002001	0.001799
t_1/2_ (min)	12.29	14.67
t_0.95_ (min)	23.35	27.88
*R* ^2^_adj_	0.9917	0.9926
SD (mg g^−1^)	0.8538	0.9551
BIC	0.56	5.04
Fractal-PFO order		
q_e_ (mg g^−1^)	23.3	27.89
k_1,0_ (min^−1^)	0.0407	0.04239
N	0.9557	0.9260
t_1/2_ (min)	11.63	13.94
t_0.95_ (min)	22.10	26.49
*R* ^2^_adj_	0.9999	0.9998
SD (mg g^−1^)	0.08003	0.1670
BIC	−92.28	−62.87
Fractal-PSO order		
q_e_ (mg g^−1^)	24.42	29.33
k_2,0_ (g mg^−1^ min^−-n^)	0.001099	0.001106
N	1.294	1.248
t_1/2_ (min)	11.93	14.31
t_0.95_ (min)	22.67	27.19
*R* ^2^_adj_	0.9975	0.9970
SD (mg g^−1^)	0.4664	0.6045
BIC	−21.78	−11.40

**Table 3:** Intraparticle diffusion and mass transfer coefficients of the adsorption of paracetamol onto MCC@Fe composite. T = 25 °C, pH 7.

	Paracetamol	
Effective diffusion coefficient D*i*/10^−8^ cm^2^ min^−1^	125 mg L^−1^	250 mg L^−1^
	0.508	0.524
Film diffusion K*_f_*/10^−4^ cm min^−1^	42.90	28.20
*Bi*	1858	1184

**Table 4:** Langmuir, Freundlich, and Liu isotherm parameters for the adsorption of PCT onto MCC@Fe material. Conditions: 30.0 mg of MCC@Fe, 20.00 mL of PCT solution, and pH 7.

Langmuir	10 °C	20 °C	25 °C	30 °C	35 °C	40 °C	45 °C
*Q*_max_ (mg g^−1^)	24.81 ± 0.33	19.49 ± 0.47	31.24 ± 1.03	31.25 ± 0.47	31.11 ± 0.13	32.63 ± 0.25	28.79 ± 0.57
*K*_L_ (L mg^−1^)	0.01644	0.06125	0.02555	0.03328	0.04453	0.05425	0.1125
*R* ^2^_adj_	0.9966	0.9747	0.9761	0.9932	0.9993	0.9999	0.9778
SD (mg g^−1^)	0.3837	0.9537	1.413	0.7480	0.2302	0.04809	1.326
BIC	−22.76	4.557	16.35	−2.735	−38.08	−85.07	14.45
Freundlich	10 °C	20 °C	25 °C	30 °C	35 °C	40 °C	45 °C
*K*_F_ (mg g^−1^ (mg L^−1^)^−1/nF^)	2.882 ± 0.53	5.408 ± 0.37	4.814 ± 1.22	5.959 ± 1.08	7.618 ± 1.05	8.759 ± 1.17	9.617 ± 0.70
*n*_F_	2.875	4.420	3.183	3.546	4.097	4.331	4.880
*R* ^2^_adj_	0.9493	0.9833	0.8790	0.9140	0.9387	0.9329	0.9755
SD (mg g^−1^)	1.485	0.7760	3.177	2.654	2.159	2.441	1.392
BIC	17.83	−1.629	40.66	35.26	29.07	32.75	15.90
Liu	10 °C	20 °C	25 °C	30 °C	35 °C	40 °C	45 °C
*Q*_max_ (mg g^−1^)	22.74 ± 0.13	25.62 ± 0.028	27.12 ± 0.028	28.92 ± 0.054	30.65 ± 0.21	32.49 ± 0.004	34.78 ± 0.01
*K*_g_ (L mg^−1^)	0.01944	0.02732	0.03230	0.03812	0.04566	0.05466	0.06541
*n*_L_	1.246	0.5122	1.705	1.330	1.054	1.017	0.5621
*R* ^2^_adj_	0.9998	0.9999	0.9999	0.9999	0.9995	0.9999	0.9999
SD (mg g^−1^)	0.1029	0.006657	0.04688	0.06945	0.1978	0.004457	0.004068
BIC	−60.74	−142.9	−84.32	−72.53	−41.12	−154.9	−157.7

**Table 5:** Thermodynamic parameters of the PCT uptake onto MCC@Fe.

Temperature (K)	283	293	298	303	318	313	318
$K_{e}^{0}$/10^3^	2.938	4.129	4.883	5.763	6.902	8.263	9.887
Δ*G*° (kJ mol^−1^)	−18.79	−20.28	−21.04	−21.81	−22.64	−23.47	−24.32
Δ*S*° (J. K^−1^.mol^−1^)	161.3 ± 2.04						
Δ*H*° (kJ mol^−1^)	27.00 ± 0.68						
*R* ^2^_adj_	0.9973						

## Data Availability

Data are contained within the article.

## Supplementary Materials

The following supporting information can be downloaded at https://www.mdpi.com/article/10.3390/polym16243538/s1, Figure S1. (A) Structural formula of acetaminophen, pKa value is indicated in the figure; (B) Optimized three-dimensional structural formula of paracetamol. Figure S2. Energy-dispersive X-ray spectroscopy of MCC@Fe magnetic composite. Figure S3. pHpzc of MCC@Fe.
