# Supplementary material for: Magnetic Composite Carbon from Microcrystalline Cellulose to Tackle Paracetamol Contamination: Kinetics, Mass Transfer, Equilibrium, and Thermodynamic Studies
Source: Polymers (Basel). 2024 Dec 19;16(24):3538. doi: 10.3390/polym16243538 (PMC11677694; doi:10.3390/polym16243538)
Supplement: Supplementary file 1 [file polymers-16-03538-s001.zip › polymers-3337699-supplementary.pdf]

Supplementary Material

2.2. One-spot preparation process of magnetic composite from microcrystalline cellulose- *MCC@Fe*

The *magnetic carbon composite* was produced by a single-route process, as described below. $FeCl_3$ was used as an activating agent to improve textural properties, precisely pore size and specific surface area, and to provide magnetic properties to the material. First, $FeCl_3$ salt was dissolved in deionized water in order to have a 0.62 mol L^{-1} solution. Afterward, 10 g of the pure *microcrystalline cellulose powder* (ϕ< 100 µm) was added to the solution and mixed thoroughly to form a paste. The mixtures were stirred up with a glass stick at 90°C for two hours [50-52]. Then, the homogeneous pastes formed were oven-dried at 105 °C overnight and carbonized in a conventional furnace (Sanchis, Brazil) [50-52]. The heating was carried out from 25° to 650°C at a heating rate of 10 °C min^{-1}, under N_2 atmosphere (flow rate of 12L h^{-1}). After reaching 650°C, the temperature was maintained for 60 minutes. The furnace was then shut down. However, the nitrogen stream was kept until the temperature reached < 200°C [33,65,66]. It is worth reminding that the magnetic property of the material was developed during the carbonization, where $FeCl_3$ is converted to Fe_3O_4. After cooling, the obtained materials were refluxed with 0.1 mol L^{-1} HCl solution at 80 °C for 120 min. Then, the carbon material was thoroughly washed with deionized water until the pH values of the washing waters attained pH 6.0 [33,41]. Finally, the materials were oven-dried at 105°C overnight [65,66]. The *magnetic biochar cellulose was labeled MCC@Fe.*

2.3. Characterization of magnetic composite carbon (MCC@Fe) adsorbent

The characterization of *MCC@Fe adsorbent was* achieved using various analytical techniques. The data of the pure cellulose microcrystalline was also provided to compare with

MCC@Fe [39]. Carbon, nitrogen, and hydrogen contents, also called elemental analysis, were obtained using an elemental analyzer (Perkin Elmer M CHNS/O model 2400). Oxygen mass fraction was calculated by subtracting the ash contents (obtained by TGA data), C, N, and H mass fraction from the total mass of the sample used for the analysis. Raw cellulose and *MCC@Fe* crystallinity were assessed using X-ray Diffraction (XRD) on a Philips X'Pert-MPD diffractometer.

Additionally, the Scherrer model [72] was employed to determine the average crystallite size of Fe_3O_4 particles in the *MCC@Fe* sample. SEM (JEOL microscope, model JSM 6060) was used to study surface morphologies of the *MCC@Fe* samples [37,40]. Chemical characterization of functional groups in bulk phase and on the surface of the *MCC@Fe* sample was achieved by FTIR (Bruker, model alpha) in the range 4000–400 cm^{-1}, using the pellets method, in which a specific amount of sample is dispersed in KBr reagent [45,52]. The SEM equipment coupled with the EDS instrument allows one to find the chemical element composition of the samples [40,45]. The textural properties represented by the calculation of the apparent surface area (S_{BET}) and pore-size distribution were evaluated based on nitrogen adsorption/desorption measurements at 77 K using a Micrometrics Instrument, TriStar II 3020 [50,51]. The thermal stability of the material was studied by TA Instruments model SDT Q600 (New Castle, USA) with a heating rate of 10°C min^{-1} at 100 mL min^{-1} of synthetic air flow. The experiment was carried out from 20° to 800 °C under N_2 gas and from 800° to 1000°C under O_2 gas [39]. Using synthetic air allows us to obtain the ash amount of the sample. The hydrophilic or hydrophobic index (HI) of the raw microcrystalline cellulose and magnetic cellulose carbon were determined as described previously [73]. The experiment was carried out in a temperature-controlled chamber at 25°C for 24h. The magnetic parameter of *MCC@Fe adsorbent* was performed using an EZ9 MicroSense VSM at ambient conditions [65,66].

2.4 Adsorption study

Preliminary adsorption experiments were performed in order to ensure the reproducibility, reliability, and accuracy. An aliquot of 20.00 mL of paracetamol (PCT) solution with the initial concentration variating from 10.0 to 500.0 mg L^{-1} was added to 50.0 mL flat-Falcon tubes with 30.0 mg *MCC@Fe* carbon material at pH ranging from 2.0 to 10.0. The Falcon tubes were capped and disposed of horizontally inside a thermostatic reciprocating agitator (Oxy 350, São Leopoldo, Brazil). The slurries were shaken at different time intervals between 1 and 300 min at 10° to 45°C with a shaking speed of 150 strikes per minute [40,41]. Subsequently, the solid phase was separated from the liquid phase using a magnet. When necessary, aliquots of 1-10 ml of the liquid phase were diluted to 1.0-25.0 mL in calibrated volumetric flasks using the blank solution. The PCT unadsorbed after the adsorption process was measured using the T90+ PG Instruments spectrophotometer at a maximum absorption wavelength of 257 nm.

The sorption capacity (Eq S1) and the percentage of adsorbate removed (Eq S2) are given below:

$$q = \frac{(C_0 - C_f)}{m}.V \quad \text{(S1)}$$

$$\% \, Removal = 100.\frac{(C_0 - C_f)}{C_0} \quad \text{(S2)}$$

q is the sorption capacity of adsorbate adsorbed by the adsorbent (mg g^{-1}). C_o is the initial adsorbate solution concentration in contact with the solid adsorbent (mg L^{-1}). C_f is the final adsorbate concentration after adsorption (mg L^{-1}). m is the mass of adsorbent (g). V is the aliquot of the adsorbate solution (L) introduced in the flask.

The study of the influence of the initial pH of adsorbate was performed at 25°C, using an initial concentration of 300 mg L^{-1} of PCT solutions, a time of contact between the adsorbent and adsorbates of 2 h, an adsorbent dosage of 1.5 g L^{-1} (n=3).

The preliminary experiments were conducted to ensure the experimental data's reproducibility, reliability, and accuracy. The relative standard deviations of all measurements (n=3) were below 3.5% [39,74]. Blanks were run in parallel and corrected when necessary [40].

The solutions of adsorbates were stored in glass bottles, cleaned, rinsed with deionized water, dried, and stored in a suitable cabinet [41].

Standard PCT solutions (2.0-50.0 mg L^{-1}) were calibrated in parallel with a blank. The linear analytical calibration curve was performed on the UV-Win software of the T90+ PG Instruments spectrophotometer. The detection limits of PCT were 0.18 mg L^{-1} with a signal/noise ratio of 3 [42].

A 15.0 mg L^{-1} of standard PCT solutions was used as quality control after every ten measurements to ensure the accuracy of the analytes measurements [45].

The kinetic and equilibrium data's fitness was done using nonlinear methods, which were evaluated using the Simplex method first and secondly by the Levenberg–Marquardt algorithm using the fitting facilities of the Microcal Origin 2021 software [39,74]. The suitableness of the kinetic and equilibrium models was evaluated using the residual sum of squares (*RSS*), the determination coefficient (R^2), the adjusted determination coefficient (R^2_{adj}), the standard deviation of residues (*SD*), and the Bayesian Information Criterion (*BIC*) [39,74]. Equations S3 to S7 are the mathematical expressions for RSS, R^2, R^2_{adj}, SD, and BIC, respectively.

$$RSS = \sum_{i}^{n} \left(q_{i,exp} - q_{i,model}\right)^2 \tag{S3}$$

$$R^2 = \left(\frac{\sum_i^n \left(q_{i,exp} - \overline{q}_{i,exp}\right)^2 - \sum_i^n \left(q_{i,exp} - q_{i,model}\right)^2}{\sum_i^n \left(q_{i,exp} - \overline{q}_{i,exp}\right)^2} \right) \tag{S4}$$

$$R^2_{adj} = 1 - (1 - R^2).\left(\frac{n-1}{n-p-1}\right) \tag{S5}$$

$$SD = \sqrt{\left(\frac{1}{n-p}\right).\sum_{i}^{n} \left(q_{i,exp} - q_{i,model}\right)^2} \tag{S6}$$

$$BIC = nLn\left(\frac{RSS}{n}\right) + pLn(n) \tag{S7}$$

In the above equations, the $q_{i,\ model}$ is the individual theoretical q value predicted by the model; $q_{i,\ exp}$ is individual experimental q value; $\overline{q}_{i,exp}$ is the average of all experimental q values measured; n is the number of experiments; p is the number of parameters in the fitting model.

The values of R^2_{adj}, SD, and BIC will be presented to compare different models of kinetics and equilibrium presented in this work. The best-fitted model would present R^2_{adj} closer to 1.000, lower values of SD, and BIC values. However, the kinetic and equilibrium models could not merely be chosen based on the values of R^2 [39,74] when these models present a different number of parameters. Therefore, it is necessary to check if the improvements in the R^2 values are due to the increase in the parameters [39,74] or if, physically, the model with more parameters better explains the process taking place [39,74].

However, the difference in BIC values between models could be conclusive if the difference in BIC values ≤ 2.0) shows no significant difference between the two models [50,55]. When the difference in BIC values is between 2 and 6, there is a positive perspective that the

model with lower BIC is the most suitable [39,74]. For variations of BIC values from 6-10, there is a strong possibility that the model with a lower BIC value would be the best model to be fitted [74]. However, if there is a difference in BIC values (10.0), it can be predicted with accuracy that the model with a lower BIC value is better fitted [74].

2.4.1 Kinetic adsorption models.

The kinetic adsorption data were evaluated by using four nonlinear models: pseudo-first-order (PFO) [74], pseudo-second-order PSO [74], fractal-like pseudo-first-order (FL-PFO) [75], and fractal-like pseudo-second-order (FL-PSO) [75]. The mathematical equations of these respective models are shown in Equations S8-S11.

$$q_t = q_e.[1 - exp\,(-k_1.t)] \tag{S8}$$

$$q_t = \frac{k_2.q_e^2.t}{1+q_e.k_2.t} \tag{S9}$$

$$q_t = q_e.[1 - exp\,(-k_{1,0}.t)^n] \tag{S10}$$

$$q_t = \frac{k_2.q_e^2.t^n}{1+q_e.k_{2,0}.t^n} \tag{S11}$$

Where t is the contact time (min); q_t, and q_e are the amount of adsorbate adsorbed at time t and the equilibrium, respectively (mg g^{-1}); k_1 is the pseudo-first-order rate constant (min^{-1}); k_2 is the pseudo-second-order rate constant (g mg^{-1} min^{-1}); $k_{1,0}$ is the fractal-like pseudo-first-order constant rate (min^{-1}), $k_{2,0}$ is the fractal-like pseudo-second-order rate constant (g mg^{-1} min^{-n}), and n is the fractional-like exponent (n >0).

2.4.2. Film and intraparticle mass transfer modeling

The knowledge of particle-liquid mass transfer coefficient (k_f) and effective diffusion coefficient (D_i) are important parameters for a rational design of a solid-liquid/stirred tank reactor (STR). The determination of the particle-liquid mass transfer coefficient (k_f) through the film surrounding a particle of the adsorbent was obtained following the method described by [76]. The transfer rate of the adsorbate in the film surrounding a spherical adsorbent particle of the adsorbent can be written as follows:

$$\frac{dq_t}{dt} = \frac{3k_f}{r\rho_p}(C_b - C_s) \quad (S12)$$

Where k_f is the film material transfer coefficient (g L^{-1}) and ρ_p is the particle density of adsorbent (g.L^{-1}). C_s and C_b are respectively the concentration (mg L^{-1}) of the adsorbate on the surface of the adsorbent particle (film-adsorbent interface) and of the adsorbate in solution. r is the radius of a particle of the adsorbent (µm). The concentration of the adsorbate (C_s) on the surface of the adsorbent is determined from the Langmuir isotherm which gives the relationship between the concentration of the adsorbate in solution and that on the surface of the adsorbent.

$$q_s = \frac{q_m.K_L.C_s}{1 + K_L.C_s} \quad (S13)$$

q_m and K_L are the the maximum adsorption capacity of Langmuir (mg g^{-1}), and equilibrium constant (L mg^{-1}), respectively. For adsorption control by film diffusion, q_s= q_t.

Therefore:

$$C_s = \frac{q_s}{K_L.(q_m - q_s)} \approx \frac{q_t}{K_L.(q_m - q_t)} \tag{S14}$$

Equation S14: Expression of the concentration of the adsorbate at the surface for the mass transfer governed by film diffusion.

For adsorption in a stirred reactor, the mass balance of the adsorbate in solution is written as follows:

$$C_b = C_0 - \frac{m.q_t}{V} \tag{S15}$$

Equation S15: Expression of the concentration of the adsorbate in solution. C_0 (mg L^{-1}) is the initial concentration of the adsorbate in an aqueous medium while m (g) is the mass of the adsorbent, and V (L) is the volume of the solution.

When equilibrium is reached, the expression of the concentration of the adsorbate in solution at equilibrium is given by:

$$C_e = C_0 - \frac{m.q_e}{V} \tag{S16}$$

$$q_e = \frac{q_m.K_L.C_e}{1 + K_L.C_e} \tag{S17}$$

From equations (S13-S17), equation (S12) becomes:

$$\frac{dq_t}{dt} = \frac{3mk_f q_m}{rV\rho_p} . \left(\frac{q_e - q_t}{q_m - q_t}\right) . \left(\frac{VC_0}{mq_e} - \frac{q_t}{q_m}\right) \tag{S18}$$

By integrating equation (S18) according to the initial conditions qt=0 to t=0, we obtain equation S19 below, which is the mass transfer rate of material into a film in the linear form:

$$\frac{3mk_f q_m}{rV\rho_p}.\left(\frac{VC_0}{mq_e}-\frac{q_e}{q_m}\right)t=-\left(1-\frac{q_e}{q_m}\right)ln\left(1-\frac{q_t}{q_m}\right)-\left(\frac{VC_0}{mq_e}-1\right)ln\left(1-\frac{mq_e}{VC_0}.\frac{q_t}{q_m}\right) \quad \text{(S19)}$$

By setting the parameter A (min^{-1}) as follows,

$$A=\frac{3mk_f q_m}{rV\rho_p}.\left(\frac{VC_0}{mq_e}-\frac{q_e}{q_m}\right) \quad \text{(S20)}$$

Equation (S19) becomes:

$$A_t=F(q_t)=-\left(1-\frac{q_e}{q_m}\right)ln\left(1-\frac{q_t}{q_e}\right)-\left(\frac{VC_0}{mq_e}-1\right)ln\left(1-\frac{mq_e}{VC_0}.\frac{q_t}{q_m}\right) \quad \text{(S21)}$$

Plotting the right-hand side of equation S21 as a function of time (t) gives a straight line with slope A. The film transfer coefficient k_f can then be deduced from the knowledge of the parameter A.

2.4.3 Particle-liquid effective diffusion coefficient (Di)

The intra-particle effective diffusion coefficient diffusion of the adsorbate in the pores of the adsorbent is described by the series de [77].

$$\frac{q_t}{q_e}=1-\frac{6}{\pi^2}\sum_{i=1}^{\infty}\frac{1}{i^2}exp(-i^2Bt) \quad \text{(S22)}$$

Where q_t et q_e are respectively the quantity of adsorbate fixed on the adsorbent for a time t and at adsorption equilibrium. i being the iteration parameter of the series while B (min^{-1}) a constant is expressed as follows:

$$B=\frac{\pi^2 D_i}{r^2} \quad \text{(S23)}$$

r, is the radius of the adsorbent particle (µm), and Di ($cm^2\ min^{-1}$), is the effective intra-particle diffusion coefficient. For a surface coverage rate less than 85%, i.e., equation S22 can be approximated by the Weber and Morris model [78].

$$Bt = F(q_t) = \left(\sqrt{\pi} - \sqrt{\pi - \pi^2 \frac{q_t}{3q_e}}\right)^2 \qquad (S24)$$

If the curve of versus the time, t, is known is a straight line, adsorption is governed by intra-particle diffusion. For a recovery rate greater than 85% of the adsorbent, equation S24 is approximated using the modified Urano and Tachikawa model

$$Bt = F(q_t) = -0.4977 - ln\left[1 - \left(\frac{q_t}{q_e}\right)\right] \qquad (S25)$$

Plotting the right-hand side of equation S25 as a function of t gives a line with slope B. Also, knowing the value of B, the intra-particle diffusion coefficient can be calculated. Knowing the film transfer coefficient k_f, and the intra-particle transfer coefficient D_i, the dimensionless numbers of Biot (Bi). In order to know which of the two transfer modes is superior to the other, the Biot number (Bi) is used. The Biot number compares the film transfer (numerator) and the intraparticle transfer (denominator). It is expressed as follows.

$$B_i = \frac{2k_f r}{D_i} \qquad (S26)$$

Where B_i is the Biot number (dimensionless), k_f is the film mass transfer coefficient, ($cm\ min^{-1}$), r is the adsorbent radius (µm) and D_i is the effective intra-particle diffusion coefficient, ($cm^2\ min^{-1}$)

Intraparticle transfer predominates over film transfer when the Biot number (Bi) is greater than 100 Consequently, the limiting step in the mass transfer process is intraparticle diffusion. When intraparticle diffusion is the limiting step, it is in fact surface diffusion that governs it. On the other hand, the mass transfer around the film surrounding the particle of the adsorbent grain predominates over intraparticle diffusion when the Biot number (Bi) is less than 100.

2.4.4 Equilibrium models.

Langmuir, Freundlich, and Liu's models were employed to analyze equilibrium data. Equations S27, S28, and S29 show the corresponding Langmuir [74], Freundlich [74], and Liu models [74].

$$q_e = \frac{Q_{max}.K_L.C_e}{1 + K_L.C_e} \quad \text{(S27)}$$

$$q_e = K_F.C_e^{1/nF} \quad \text{(S28)}$$

$$q_e = \frac{Q_{max}.(K_g.C_e)^{nL}}{1+(K_g.C_e)^{nL}} \quad \text{(S29)}$$

Where q_e is the adsorbate amount adsorbed at equilibrium (mg g^{-1}); C_e is the adsorbate concentration at equilibrium (mg L^{-1}); Q_{max} is the maximum sorption capacity of the adsorbent (mg g^{-1}); K_L is the Langmuir equilibrium constant (L mg^{-1}); K_F is the Freundlich equilibrium constant [mg.g^{-1}.(mg.L^{-1})$^{-1/nF}$]; K_g is the Liu equilibrium constant (L mg^{-1}); n_F and n_L are the exponents of Freundlich and Liu model, respectively, (n_F and n_L are dimensionless).

2.4.5 Adsorption thermodynamics

Thermodynamic studies for the adsorption of PCT onto *MCC@Fe* material were performed at temperatures ranging from 10ºC to 45°C (283 to 318K).

The Gibb's free energy change ($\Delta G°$, kJ mol^{-1}), enthalpy change ($\Delta H°$, kJ mol^{-1}), and entropy change ($\Delta S°$, J mol^{-1}K^{-1}) were evaluated with the aid of Equations S30-S34, respectively [74,80].

$$\Delta G^0 = \Delta H^0 - T.\Delta S^0 \quad \text{(S30)}$$

$$\Delta G^0 = -RT.LnK_e^0 \quad \text{(S31)}$$

$$K_e^0 = \frac{(1000.K_g.Mw.[adsorbate]^0}{\gamma} \quad \text{(S32)}$$

The combination of Equations S30 and S31 leads to equation S33

$$LnK_e^0 = \frac{\Delta S^0}{R} - \frac{\Delta H^0}{R}.\frac{1}{T} \quad \text{(S33)}$$

The nonlinear form of equation S33 is [74]:

$$K_e^0 = exp\left[\frac{\Delta S^0}{R} - \left(\frac{\Delta H^0}{R}\right).\frac{1}{T}\right] \quad \text{(S34)}$$

Where R is the universal gas constant (8.314 J K^{-1} mol^{-1}); T is the absolute temperature (Kelvin); ^{0} is the thermodynamic equilibrium constant, which was calculated according to equation S32. ^{0} is dimensionless.

$K_e{}^0$ is calculated by converting the values of K_g (Liu equilibrium constant) or K_L (Langmuir equilibrium constant), which is expressed in L mg^{-1} into L mol^{-1}. Firstly, the value K_g or K_L is multiplied by 1000 (mg g^{-1}), and then multiplied by the molecular weight of the adsorbate (g mol^{-1}) and by the standard concentration of the adsorbate (1 mol L^{-1}) and divided by the activity coefficient of the adsorbate (dimensionless) [74,80]. The solution is assumed to be sufficiently diluted to consider that the activity coefficient is unitary. Making these calculations, ^{0} becomes dimensionless [74,80].

Equation S33 was used for calculating ΔH° and ΔS°, and Equation S31 was used for calculating ΔG°.

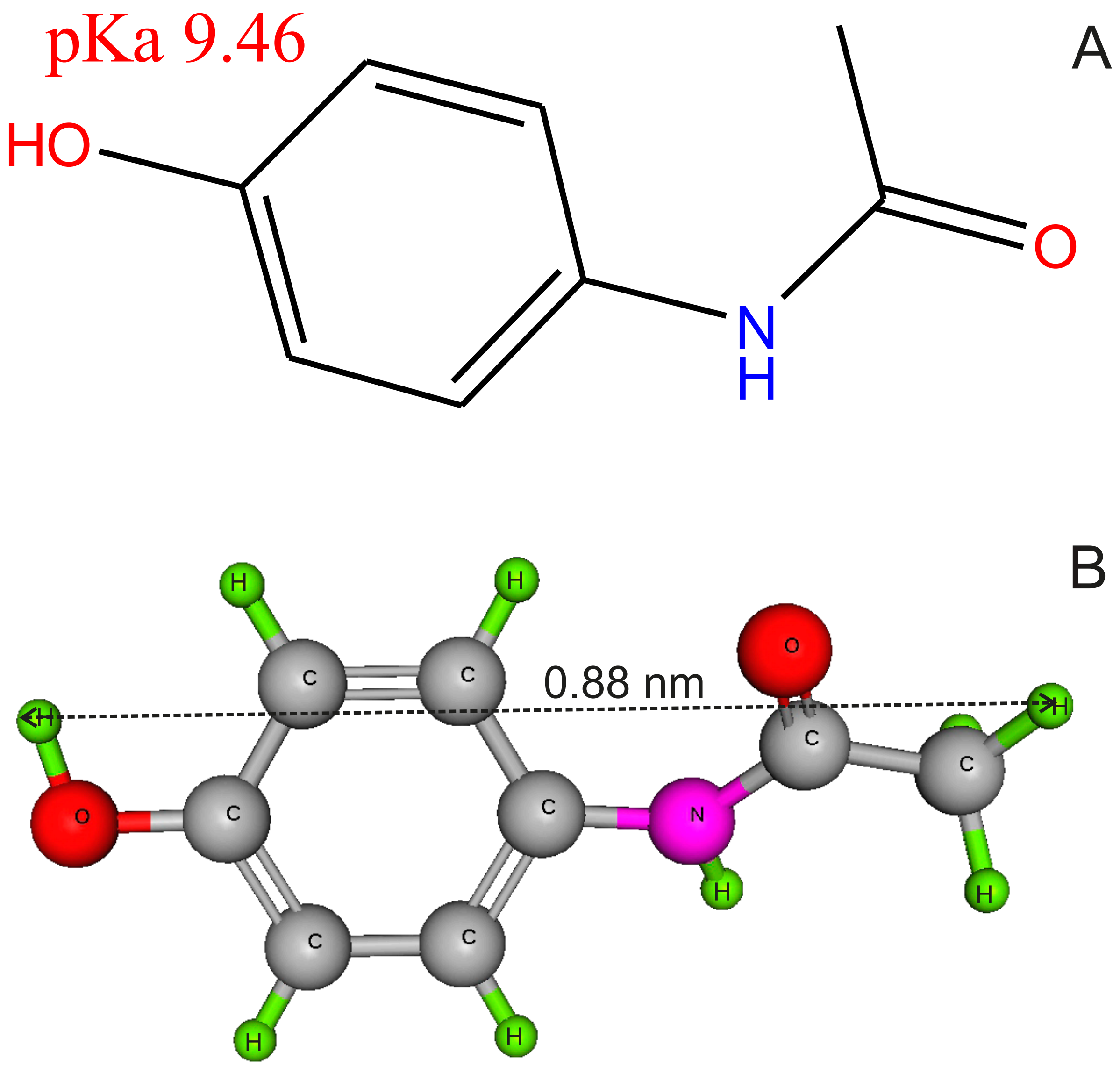

Fig S1. A) Structural formula of acetaminophen, pKa value is indicated in the figure; B) Optimized three-dimensional structural formula of paracetamol. The dimensions of the chemical molecule was calculated using MarvinSketch version 24.1.2. van der Waals surface area 226.76 Å^2 (pH 7.0); Polar surface area 49.33 Å^2 (pH 7.0); van der Waals volume 138.0 Å^3, Dipole Moment 3.47 Debye; LogP 0.46; Hydrophilic-lipophilic balance 11.12-16.15

Chemical elements	Containing (%)
C	62.31
O	14.66
Fe	2.81

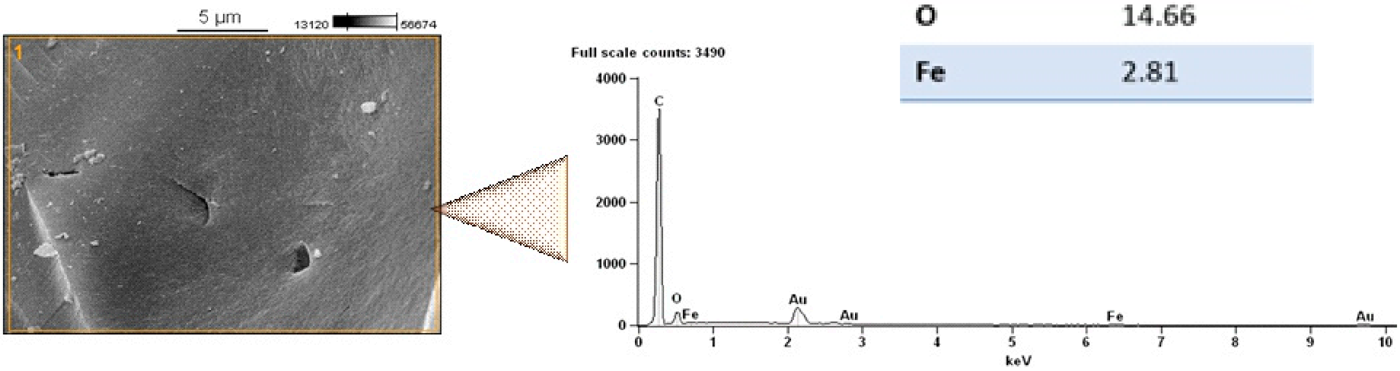

Fig S2. Energy-dispersive X-ray spectroscopy of MCC@Fe magnetic composite

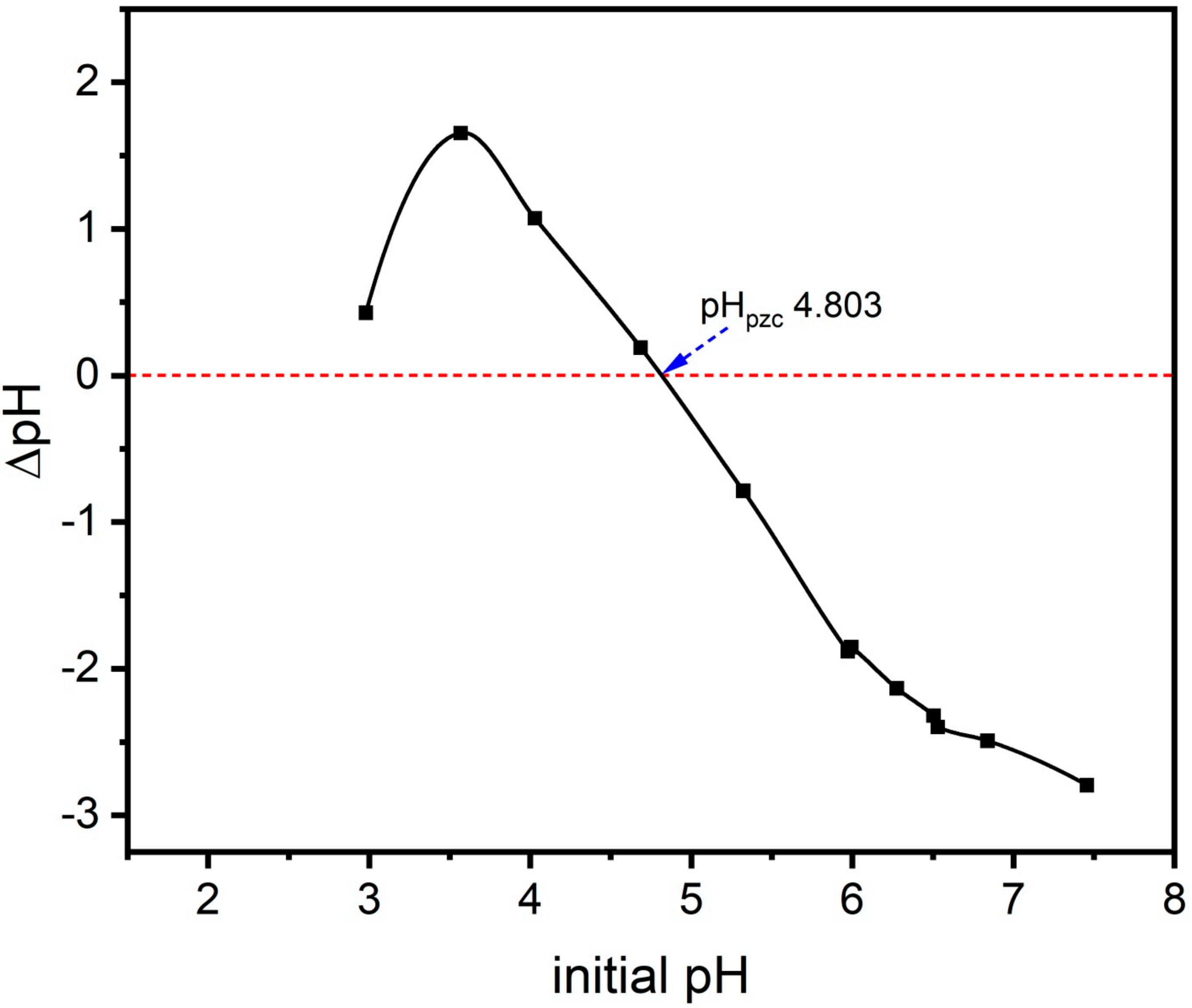

Fig S3. pH$_{pzc}$ of MCC@Fe
